# The use of tensiomyography in older adults: a systematic review

**DOI:** 10.3389/fphys.2023.1213993

**Published:** 2023-06-16

**Authors:** Katarina Pus, Armin H. Paravlic, Boštjan Šimunič

**Affiliations:** ^1^ Science and Research Centre Koper, Institute for Kinesiology Research, Koper, Slovenia; ^2^ Faculty of Sport, University of Ljubljana, Ljubljana, Slovenia; ^3^ Department of Health Sciences, Alma Mater Europaea—ECM, Maribor, Slovenia; ^4^ Faculty of Sports Studies, Masaryk University, Brno, Czechia

**Keywords:** muscle function, elderly, neuromuscular function, normative values, TMG

## Abstract

**Introduction:** Aging of skeletal muscles results in a cascade of events negatively affecting muscle mass, strength, and function, leading to reduced mobility, increased risk of falls, disability, and loss of independence. To date, different methods are used to assess muscle mechanical function, tensiomyography (TMG) being one of them. The aim of this review was twofold: to summarize the evidence-based usefulness of tensiomyography in older adults and to establish reference values for the main tensiomyography parameters in older adults.

**Methods:** The PubMed, Web of Science, SPORTDiscus, and tensiomyography databases were searched from inception until 25 December 2022. Studies investigating older adults (aged 60+ years) that reported tensiomyography-derived parameters such as contraction time (Tc) and/or maximal displacement (Dm) were included. Methodological quality was assessed using the Quality Assessment Tool for Observational Cohort and Cross-Sectional Studies.

**Results:** In total, eight studies satisfied the inclusion criteria. Tensiomyography has been used on different groups of older adults, including asymptomatic, master athletes, patients with peripheral arterial disease, and patients with end-stage knee osteoarthritis with a mean age of 71.5 ± 5.38 (55.7% male subjects). The most evaluated were leg muscles such as vastus lateralis (VL), gastrocnemius medialis (GM), and biceps femoris (BF). The present review demonstrates that tensiomyography is used to assess neuromuscular function in asymptomatic and diseased older adults. When compared to asymptomatic individuals, power master athletes, knee osteoarthritis patients, and patients diagnosed with peripheral arterial disease have the shortest Tc in BF, VL, and GM muscles, respectively. On the other hand, endurance master athletes showed the longest Tc in all three evaluated muscles. Less mobile, nursing-home residents showed higher Dm in VL and BF, while lower Dm in GM than the asymptomatic group. The knee osteoarthritis group showed the largest Dm in BF and VL while having the smallest Dm in GM.

**Conclusion:** Tensiomyography can serve as a valuable tool for assessing neuromuscular function in older adults. The method is sensitive to muscle composition, architecture, and (pre) atrophic changes of the skeletal muscles and might be responsive to muscle quality changes in aging and diseased populations.

**Systematic Review Registration:** https://www.crd.york.ac.uk/prospero/display_record.php?RecordID=402345, identifier CRD42023402345.

## 1 Introduction

Skeletal muscle aging is a complex process that is characterized by a decline in muscle mass (Nilwik et al., 2013), strength (Edwén et al., 2014), and function (Wilkinson et al., 2018). Loss of muscle mass can be most likely explained by muscle fiber atrophy and loss of motor units. Although this decline is a normal part of the aging process, it can lead to decreased mobility and increased risk of falls, disability, loss of independence, and consequently, poor quality of life (Inouye et al., 2007; Woo et al., 2016; Shafrin et al., 2017). Understanding the factors that contribute to muscle aging is important for developing the most effective interventions to maintain optimal muscle function and promote healthy aging. On the other hand, it is essential to be cautious when reporting trends in muscle decline associated with the aging process. It can be difficult to ascertain the degree of the decline attributable solely to the aging process itself, as opposed to declining muscle mass due to other associated risk factors, such as lifestyle changes that often accompany aging, such as reduced physical activity or increased physical inactivity. As such, we must consider all the contributing factors when looking at this important issue. To support this, older adults are the most sedentary and least physically active population (De Rezende et al., 2014), typically engaging in an average of 9.4 h of sedentary behavior per day (Harvey et al., 2015). From strictly controlled bed rest studies, we have learned that physical inactivity, when imposed on older adults, decreases skeletal muscle mass and function (on muscle fiber and whole muscle levels) to a larger extent than in the younger counterparts (Pišot et al., 2016; Rejc et al., 2018). In addition, older participants have a slower or even incomplete recovery than younger participants after 14 days of bed rest (Pišot et al., 2016; Rejc et al., 2018). Therefore, it is essential and of great clinical importance to assess skeletal muscle atrophy-related function before atrophy occurs in older persons.

One group that has received particular attention in this regard is master athletes, who are individuals over the age of 35 years (lower limits depend on sports) who engage in competitive sports. Therefore, they tend to maintain their lifelong physical and sports-related activity levels and have higher muscle mass and function levels compared to sedentary individuals of the same age (Mckendry et al., 2018). For example, non-athletes show a progressive decline of muscle mass of approximately 3%–8% per decade after the age of 30, and this rate of decline is even higher after the age of 60 years (Holloszy, 2000; Melton et al., 2000). This suggests that regular physical activity may be able to mitigate some of the negative consequences aging has on the skeletal muscles. It has been found that muscle strength and function decline even more when coupled with physical inactivity (Di Girolamo et al., 2021; Müller et al., 2021). To support the importance of physical activity in preserving muscle structure, it was shown that muscle mass declines as much as 4.5% after only 7 days of bed rest in young adults (Pišot et al., 2016; Šimunič et al., 2019). When comparing master athletes to non-athletes, master athletes maintain 12% greater leg lean mass and 17% lower body fat percentage at the age of ∼70 years (Piasecki et al., 2019), suggesting the importance of preserving high levels of physical activity across a life-span.

Muscle mechanical function can be assessed by different means. Dynamometry is used most often, whereas other physical performance tests such as timed up-to-go, sit-to-stand, and 4-m walking speed-for-time tests were commonly used among older adults. These are included in several diagnostic criteria for classifying pathologies like sarcopenia (Cruz-Jentoft et al., 2019) or Parkinson’s disease (Paschal et al., 2006). The original operational definition of sarcopenia was based solely on low muscle mass (Cruz-Jentoft et al., 2010). However, in 2010, the European Working Group on Sarcopenia in Older People (EWGSOP) issued guidelines that classified sarcopenia based on three consecutive criteria in the following order: low muscle quantity, low muscle strength, and low muscle performance (Cruz-Jentoft et al., 2010). In 2019, the same group revised the guidelines for sarcopenia classification, and muscle strength was recognized as a superior predictor for adverse outcomes than muscle mass (Schaap et al., 2013; 2018; Leong et al., 2015). Thus, the consecutive criteria were rearranged according to it (in that order): low muscle strength, low muscle quantity or quality, and low muscle performance (Cruz-Jentoft et al., 2019). The aforementioned term muscle quality was defined as the criteria to be sensitive to describe micro- and macroscopic aspects of muscle architecture and composition. However, because of technical limitations, muscle quality remains challenging as the primary parameter to define sarcopenia—a reason an “or” was used in the aforementioned protocol description (Cruz-Jentoft et al., 2019).

A promising tool for the non-invasive assessment of muscle quality is tensiomyography (TMG). It is a mechanomyography method that assesses superficial skeletal muscle’s belly radial displacement and muscle fiber oscillation after single electrical pulse stimulation in isometric conditions (twitch). TMG requires a relatively simple setup; it is mobile and measures several contractile parameters: contraction time (Tc), peak radial maximal displacement (Dm), delay time (Td), half-relaxation time (Tr), sustain time (Ts), and radial contractile velocity (Vc) (Šimunič et al., 2011; Šimunič et al., 2019). It was demonstrated that TMG-derived contractile parameters (Td, Tc, and Tr) are highly associated with skeletal muscle composition (Šimunič et al., 2011). Dm was found to be negatively correlated with muscle thickness loss after a 35-day bed rest period (Pišot et al., 2008). Moreover, a recent study showed that Dm increases already after a few days of bed rest when muscle diameter loss was not confirmed using standard clinical tools. The same study suggested that Dm can be a measure of the earliest onset of muscle decline when atrophy does not yet occur (Šimunič et al., 2019).

Despite the growing literature on the use of TMG, to the best of the authors’ knowledge, there is no summarized evidence about its use and feasibility among older adults. Therefore, the aim of this review was twofold: a) to summarize the evidence-based usefulness of the TMG in older adults and b) to establish reference values for the main TMG parameters in older adults.

## 2 Methods

### 2.1 Search strategy

This study was registered in the international prospective register of systematic reviews (PROSPERO) on 6 March 2023 (registration number CRD42023402345).

The Preferred Reporting Items for Systematic Reviews and Meta-Analyses (PRISMA) were followed for performing the present review (Page et al., 2021). Two authors (KP and BŠ) independently performed a literature search from inception to 25 December 2022 of the PubMed, Web of Science, and SPORTDiscus electronic databases. In addition, on 26 December 2022, the authors conducted a thorough search of the TMG-BMC website (TMG-BMC Ltd, 2022) to find additional articles. The following terms and their combinations were used as a search strategy string: (a) target population: “elderly,” “older adults,” “senior,” “geriatric,” and “aging”; (b) intervention: “tensiomyography”, and “TMG”; (c) comparison indicated; (d) outcome corresponded to tensiomyographic assessment. Inclusion criteria consisted of samples older than 60 years with no restriction to sample size and papers written in English. The authors also consulted other experts in the field to identify any additional published studies.

### 2.2 Eligibility criteria

Eligible studies were selected based on the PICOS criteria and were defined as follows: “P” (population) corresponded to people older than 60 years and of any gender or ethnicity; “I” (intervention) corresponded to the use of TMG; “C” (comparison) was not used; “O” (outcome) corresponded to TMG-derived parameters; and “S” (study design) selected all study types except case studies and reviews. Overall, the selected studies focused on changes in TMG-derived parameters.

### 2.3 Study selection and data extraction

Titles and abstracts from the electronic searches were screened independently by two authors (KP and BŠ). The full texts of selected articles were then checked by the same two authors to consider the fit with eligibility criteria. In case of any disagreement between the authors, a third reviewer (AP) was consulted to make a final decision. An electronic database Rayyan was used to store all relevant data. Data were extracted separately by two authors (KP and BŠ). In the case of disagreement, the third author (AP) cross-examined doubtful data. The following data were extracted: authors, year of publication, study population (sample size, gender, and age), subjects’ characteristics, and TMG-derived parameters. In case of missing data, the authors of the publications were contacted. We have used data for a dominant (kicking) leg for the analysis. If data for both legs were reported, an average was used for the analysis. For each group, a weighted averaging technique was used to obtain the pooled average and standard deviation of Tc and Dm. Consequently, an article with a greater sample size contributed more to the pooled values. Lastly, the NHLBI-NIH scale was used to determine the methodological quality of studies (NLHBI, 2021).

### 2.4 Methodological quality assessment

Two authors (KP and BŠ) independently assessed methodological quality using the Quality Assessment Tool for Observational Cohort and Cross-Sectional Studies published by the National Heart, Lung, and Blood Institute (NHLBI). This tool consists of 14 items, each of which could be marked as Yes, No, or Not Reported/Not Applicable. A score of 1 assigns to Yes, and a score of 0, to all other answers. The total score would be the number of affirmative responses. For qualitative evaluation of the final scores, scores higher than 12 were deemed good, those lower than 9 were considered weak, and those that fell in the range of 9–12 represented fair studies.

## 3 Results

### 3.1 Study selection and characteristics

**FIGURE 1:**
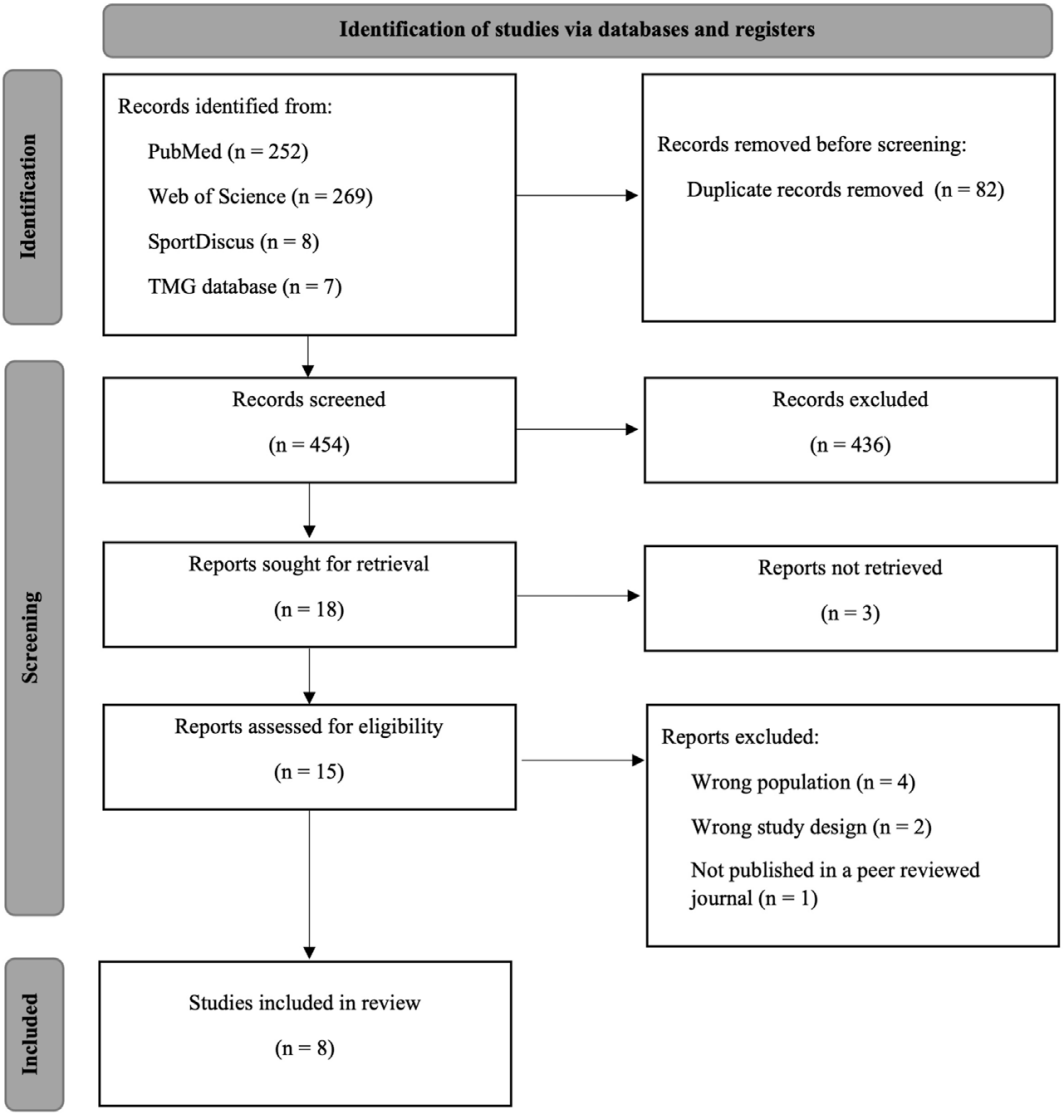
PRISMA flow diagram of included studies.

A total of 529 articles were identified in the initial search across the databases, and seven additional articles were found through other sources (Figure 1). After duplicate removal, 454 articles remained. After title and abstract screening, 436 records were excluded. The full texts of 17 articles were assessed for eligibility; however, three articles were not retrieved.

Each article was coded for study characteristics, participant information, TMG-derived parameters of Tc and Dm, and study outcomes (Table 1). Four articles reported an inappropriate population for this review, one was a case study, and another one was a review article; therefore, they were excluded. Finally, eight articles, published from 2012 until 2022, were included in this systematic review with a total of 510 participants (55.7% male subjects) with a weighted mean age of 71.5 ± 5.38.

**TABLE 1:** Characteristics of included studies.

Author	Year	Study design	Considered sample		Age (mean ± SD)	Muscles measured	Reported parameters	Mean methodological quality score
			N (% of male subjects)	Health status				
Gasparini et al.	2012	Cross-sectional	17 (82%)	Peripheral arterial disease patients	62.7 ± 10.3	GM	Tc, Dm, and Vc	9
Rodríguez-Ruiz et al.	2013	Cross-sectional	21 (100%)	Asymptomatic individuals	72.4 ± 4.93	BF and VL	Vrn	8.5
Šimunič et al.	2018	Cross-sectional	321 (38%)	Master athletes and asymptomatic individuals	71.95 ± 5.13	BF, GM, and VL	Tc	8
Zubac et al.	2019	Controlled intervention	23 (35%)	Asymptomatic individuals	66.8 ± 5.1	BF, GL, GM, TA, and VL	Tc and Dm	12
Paravlic et al.	2020	Controlled intervention	26 (54%)	Patients after knee arthroplasty	61.12 ± 5.34	BF, GM, RF, VL, and VM	Td, Tc, Dm, Ts, and Tr	10.5
Fabiani et al.	2021	Cross-sectional	28 (0%)	Nursing home residents	85.6 ± 7.6	BF, GM, VL, and VM	Tc and Dm	7.5
Ziegl et al.	2021	Cross-sectional	23 (0.04%)	Nursing home residents	86.74 ± 7.88	BF, GM, VL, and VM	Td, Tc, and Dm	9.5
Jacob et al.	2022	Cross-sectional	51 (35%)	Asymptomatic individuals	64.1 ± 3.3	VL	Tc and Dm	9

Tc, contraction time; Dm, maximal amplitude; Vc, radial contraction velocity; Vrn, normalized radial contraction velocity; BF, biceps femoris; GL, gastrocnemius lateralis; GM, gastrocnemius medialis; RF, rectus femoris; TA, tibialis anterior; VL, vastus lateralis; VM, vastus medialis.

The minimum sample size per study was 17 (Gasparini et al., 2012), while the maximum was 150 (Šimunič et al., 2018). The most measured muscles were the gastrocnemius medialis (GM), vastus lateralis (VL), and biceps femoris (BF); however, some articles also report data for semitendinosus, rectus femoris, vastus medialis, gastrocnemius lateralis, and tibialis anterior. Two included studies report an exercise intervention: plyometric jumping (Zubac et al., 2019) and strength training after total knee arthroplasty (Paravlic et al., 2020). The most reported TMG parameters were Tc and Dm; one of the included articles only reported Vc (Rodríguez-Ruiz et al., 2013).

### 3.2 Data extraction and methodological quality assessment

Two authors (KP and BŠ), blinded to each other’s results, screened the title and abstracts in accordance with inclusion criteria using the Rayyan web application. Afterward, full-text articles were screened by the same authors. In case of disagreement, the third author (AP) was consulted, who independently rated methodological quality. The median NIH score was 9, with values ranging from 6 to 12, suggesting that the included studies were generally of fair quality.

### 3.3 Tc in different groups

**FIGURE 2:**
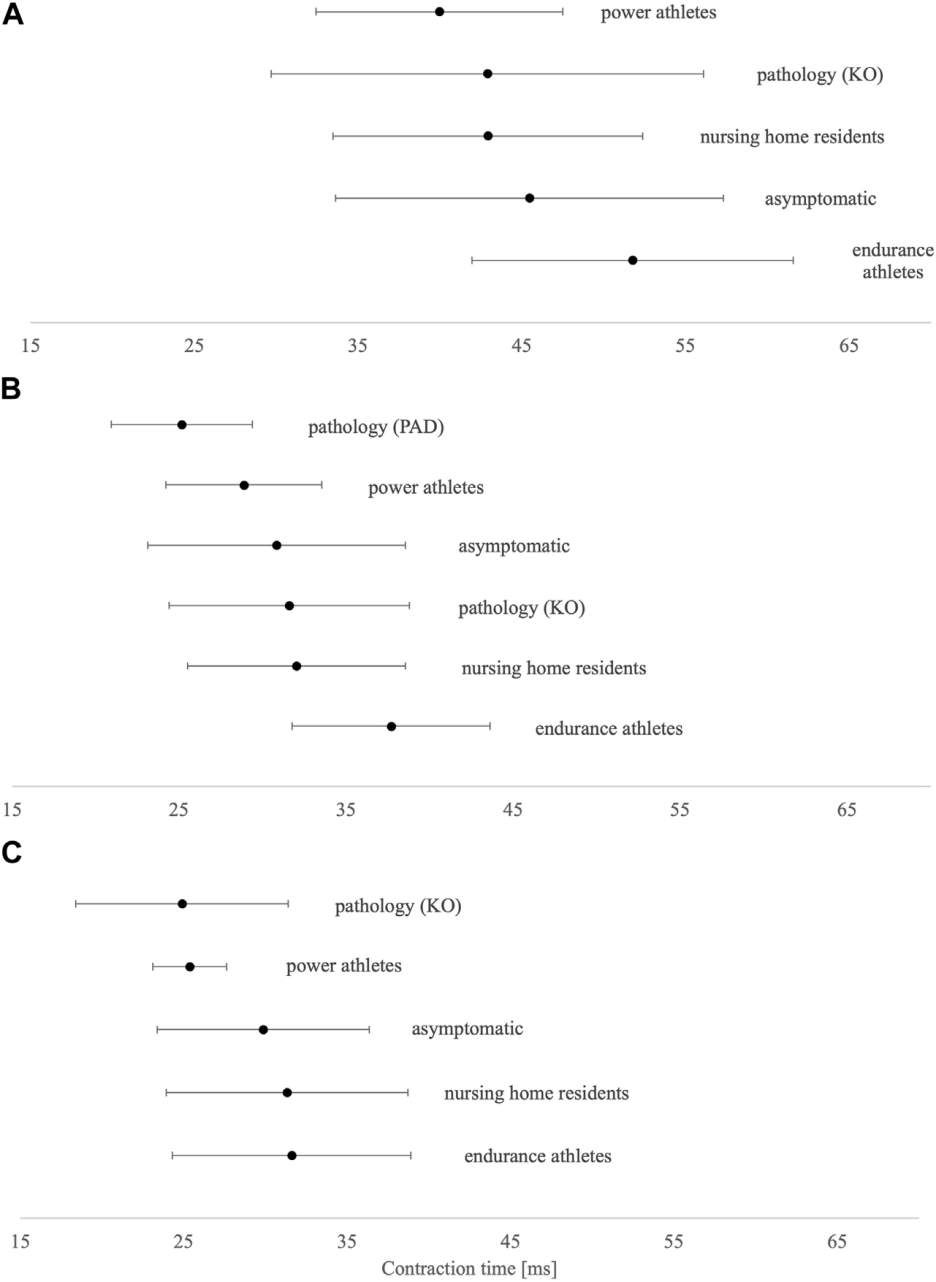
Tc means and SD results of **(A)** biceps femoris, **(B)** gastrocnemius medialis, and **(C)** vastus lateralis. KO, knee osteoarthritis; PAD, peripheral arterial disease.

In the asymptomatic group, Tc was shorter in both postural muscles VL and GM (∼30 ms) than in non-postural GM (∼46 ms) (Figures 2A–C). Compared to asymptomatic individuals, power master athletes, knee osteoarthritis patients, and peripheral arterial disease patients have the shortest Tc in BF, VL, and GM muscles, respectively. On the other hand, endurance master athletes have the longest Tc in all three presented muscles.

### 3.4 Maximal radial Dm in different groups

**FIGURE 3:**
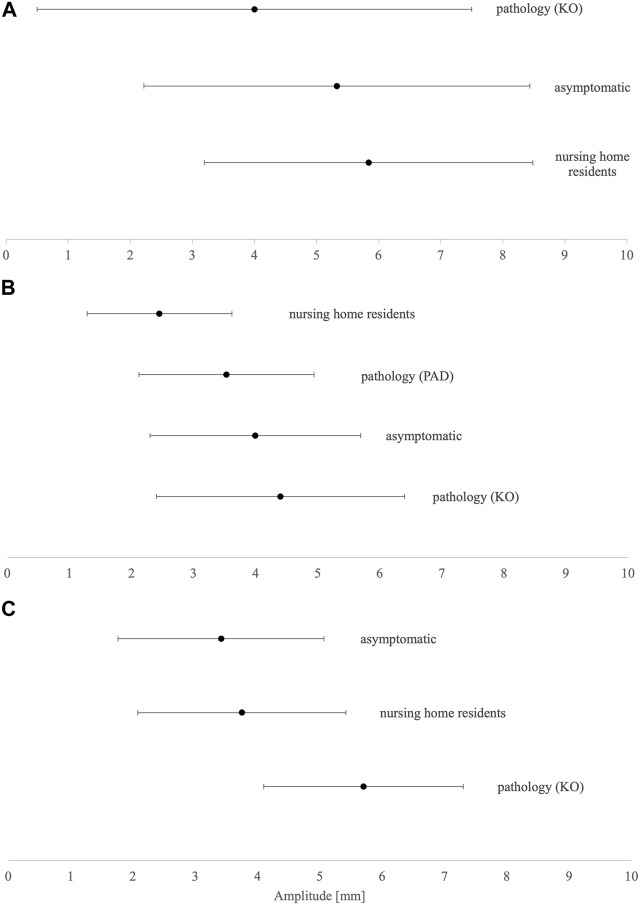
Dm means and SD results of **(A)** biceps femoris, **(B)** gastrocnemius medialis, and **(C)** vastus lateralis. KO, knee osteoarthritis; PAD, peripheral arterial disease.

The summarized data were less clear for Dm (Figures 3A–C). The less mobile, nursing home resident group has higher Dm in VL and BF, while lower Dm in GM than the asymptomatic group. The knee osteoarthritis group has the highest Dm in BF and VL while having the lowest in GM. However, peripheral arterial disease patients have Dm data only for GM, which is higher than in nursing home residents and smaller than in the asymptomatic group.

## 4 Discussion

The primary aim of the review was to summarize the evidence-based usefulness of the TMG device in older adults and to provide some future directions for the use of TMG in older adults. The secondary aim was to establish reference values for the main TMG parameters measured in older adults. We identified a total of eight eligible studies investigating contractile parameters of a muscle using TMG.

### 4.1 Populations assessed and aims of original studies

The only included study investigating aging *per se* was the study of Šimunič et al. (2018), who studied master athletes. Master athletes maintain high levels of physical activity (Hawkins et al., 2003; Rittweger et al., 2004; Suominen and Korhonen, 2010) and suffer from fewer morbidities than non-athletes (Kettunen et al., 2006), thereby providing a unique human research model to disentangle the effects of disuse and comorbidities from aging. Šimunič et al. (2018) cross sectionally compared Tc of GM, BF, and VL in power- and endurance-master athletes from track and field disciplines with non-athletes in the age groups of 35–50, 50–65, and 65+ years. A pooled sample consisting of both men and women confirmed shorter Tc in power master athletes (both sexes and all three muscles) and longer Tc in endurance master athletes than in non-athletes, respectively. This was further confirmed with a higher proportion of type I muscle fibers in endurance master athletes and a higher proportion of type IIx muscle fibers in power master athletes. Interestingly, the authors pointed out the non-linear slowing of Tc in endurance master athletes after the age of 65 years, which warrants further explanation. Although the increase in Tc was very consistent in the BF muscle, it was less evident in both postural muscles, i.e., GM and VL, as these muscles experience sufficient habitual load for maintaining their contractile properties. To support this, Jacob et al. (2022) investigated age-related VL contractile properties in healthy adults aged between 18 and 70. They concluded that the Tc of the pooled sample increases beyond the age of 50, but neither male nor female subjects could corroborate this rise with age. However, they found a reduction of Dm after the age of 50 years, with some evident differences in male versus female subjects. It was shown that male subjects experience a decrease in Dm after the age of 50 years, whereas in female subjects, this occurs after 60 years of age. Another study conducted by Rodriguez-Ruiz et al. (2013) investigated the effects of age and physical activity on normalized response velocity in VL and BF muscles by comparing four age groups (teenage, undergraduate, adult, and older adults). Compared to reference values of the teenage group, a decline in VL Vc was already evident in undergraduate students (mean age 22.8 ± 1.99 years); however, BF Vc showed a decline only in the group of older adults (mean age 72.4 ± 4.93 years). Although this is the only study reporting VL Vc, we believe the methodological approach in its estimation is questionable and warrants further investigation. Specifically, they estimated normalized response velocity by dividing a value of 0.8 with a Tc yielding a result in ms^−1^. However, the authors also provided us with Tc and maximal Dm data that were used for our secondary aim—reference values (see the following paragraph). The only longitudinal randomized control trial was performed by Zubac et al. (2019), who studied the effects of 8-week plyometric exercise on explosive power and TMG parameters of five muscles. Improvements in explosive power were paralleled by shorter Tc in BF and GM (not in VL, GL, and TA), and lower Dm was found only in BF.

TMG has been used in clinical studies with different pathologies. Gasparini et al. (2012) investigated peripheral arterial disease patients with intermittent claudication that was persistent for a minimum of 3 months. More specifically, the authors evaluated the fatigability of GM muscle in peripheral arterial disease patients and the asymptomatic control group. In comparison to asymptomatic controls, patients presented shorter Tc and were fatigued earlier. In addition, patients showed a different fatigue curve pattern than that observed in controls, evidenced by shortened Tc during the first 30 s before gradually increasing back to the initial level and beyond. At the same time, Dm exhibited a peak increase after approximately 30 s, followed by a gradual decrease to the initial level. In comparison, the asymptomatic control group demonstrated only a shortening of Tc and increase of Dm during the same 1-min continuous 1-Hz electrical stimulation of the GM muscle. Moreover, the authors proposed calf muscle biomechanical function to be considered when prescribing therapies for walking performance improvements for peripheral arterial disease patients. Paravlic et al. (2020) investigated the effects of total knee arthroplasty on muscle-specific contractile properties and function. They found that in the early post-surgery phase, contractile properties of the muscles surrounding the knee joint have been negatively affected, especially VM, where Dm decreased 1-month post-surgery and indicated increased muscle stiffness. The observed results might indicate the existence of arthrogenic muscle inhibition induced by chronic osteoarthritis and subsequent surgical trauma.

Studies conducted by Fabiani et al. (2021) and Ziegl et al. (2021) have shed light on the potential link between sarcopenia physical criteria tests, like timed up-and-go and walking ability, and TMG-derived parameters in nursing home older female individuals. More specifically, longer Tc in VL and lower Dm in BF and VM were associated with lower test performance. Similar results were obtained by Ziegl et al. (2021), where they investigated the link between subtasks of timed up-and-go and TMG-derived parameters in BF, GM, VM, and VL. They found a correlation between Tc and all of the timed up-and-go subtasks, except the turning phase. Again, lower Dm has been associated with reduced mobility, which can be attributed to changes in muscle tissue brought on by physical inactivity and/or aging. Studies investigating the effects of aging or reduced physical activity on muscle structure support latter results by reporting obvious infiltration of non-contractile tissues within the muscles, such as intramuscular fat or connective tissues, being coupled with impaired excitation–contraction coupling (Tieland et al., 2018). As the EWGSOP is proposing a quest for muscle quality biomarkers that should be sensitive to describe micro- and macroscopic aspects of muscle architecture and composition (Cruz-Jentoft et al., 2019), TMG-derived parameters are excellent candidates as they are sensitive to sarcopenia criterion tests (Fabiani et al., 2021; Ziegl et al., 2021), and to muscle composition (Šimunič et al., 2011) and architecture (Pišot et al., 2008; Šimunič et al., 2019; Franchi et al., 2022).

In summary, TMG has been used to examine the effects of “pure” aging in a unique population of master athletes, to evaluate physical exercise in two pathological patients undergoing rehabilitation, and in a sarcopenic-less mobile group of institutionalized older people.

### 4.2 Differences in Tc parameters according to the population assessed

Shortest Tc was found in power master athletes (BF) and both pathological groups (VL and GM), whereas longest Tc was consistently found in endurance-trained master athletes in all three muscles. Shorter Tc (also Td and Tr) in VL correlates with a lower proportion of MHC I proportion (Šimunič et al., 2011), confirming previous results (Dahmane et al., 2001). Therefore, it seems that anaerobic sports are promoting fast-twitch muscle fibers in aging. This was implied by Šimunič et al. (2018), whose study showed differences in MHC distribution between groups of power- and endurance-trained master athletes being paralleled to Tc differences. However, when investigating athletic populations through cross-sectional studies, we must consider if found differences are due to the selection process or due to training specificity. To support the second paradigm, a TMG was used to longitudinally follow young athletes and non-athletes from 9 to 14 years of age (Šimunič et al., 2017). Athletes differentiate from non-athletes with lower Tc in VL and BF no sooner than at the age of 12 years. Similar to the youth population, studies within adults have shown that resistance training induces changes in muscle fiber type composition, favoring type II muscle fibers (Sharman et al., 2001) and increasing the muscle cross-sectional area (Liu et al., 2003; Verdijk et al., 2009).

Peripheral arterial disease patients are known to have an increased proportion of fast-twitch muscle fibers due to chronic hypoxic conditions in leg muscles (Schieber et al., 2017; McDermott et al., 2020). This condition is most often shown through peri intermittent claudication, where ischemia is present due to decreased arterial blood flow in lower-limb muscles. The calf muscles of PAD patients have different contractile characteristics in comparison to healthy individuals, which was also confirmed by our results. This could be explained by underlying disease mechanisms that alter muscle control characteristics, resulting in faster contractions, which happen due to the predominance of type II muscle fibers, eventually inducing lower resistance to muscle fatigue. Gasparini et al. (2012) found shorter Tc and increased fatigability in GM of peripheral arterial disease patients. Latter results are in line with previous studies conducted by McGuigan et al. (2001) and Ambrosio et al. (2000), who found that the GM muscles of PAD patients have decreased expression of MHC I isoforms and increased expression of MHC IIb and MHC IIx isoforms. Repeated episodes of ischemia trigger a shift from slow-twitch to fast-twitch fibers, the last being subject to earlier energy depletion and muscle fatigue. The impact of peripheral arterial disease on muscle phenotype is clearly an important area of research, and there is a need to establish what effect these pathophysiological alterations might have on the quality of life.

Patients after total knee arthroplasty and power master athletes presented the shortest Tc in VL. In patients awaiting total knee arthroplasty, Noehren et al. (2018) found −32% lower quadriceps strength, −11.6% lower proportion of VL type I fibers, and 8.2% higher proportion of VL type IIa/x hybrid fibers than that in healthy controls. Muscle fiber type composition is an indicator of global muscle health (Pette and Staron, 1997). In general, the presence of “hybrid” muscle fibers co-expressing type IIa/x MHC is indicative of poor muscle health (Pette and Staron, 1997; Tanner et al., 2002) and may contribute to impaired muscle function. Limited work in orthopedic populations indicates that knee joint injury results in unfavorable slow-to-fast fiber-type transitions in the VL that are not reversed after rehabilitation (Noehren et al., 2016). To support this, in people with advanced-stage knee osteoarthritis, a high prevalence of hybrid fibers has been reported in the VL (Callahan et al., 2015; Noehren et al., 2018); however, the precise mechanisms or causality that mediate fiber-type transitioning are still being explored.

Knowing that endurance master athletes possess a higher proportion of type I and IIa VL fibers than power master athletes and non-athletes (Korhonen et al., 2006; Pollock et al., 2018), it has been anticipated that Tc is higher in all three muscles for both sexes and all age groups. However, we did not expect a disproportional increase of Tc after the age of 65 years to be consistent across sex and age groups. The potential involvement of myosin glycation was proposed as the underlying mechanism of observed results, but this speculation also warrants further investigation (Goodpaster, 2001; Šimunič et al., 2018).

Overall, it seems that TMG-derived Tc is a sensitive and non-invasive muscle biomechanical parameter reflecting the skeletal muscle composition. Although Šimunič et al. (2011) provided a regression model for the VL muscle, there is no doubt the principles (not a regression model equation) might also apply to other muscles and studied populations.

### 4.3 Differences in Dm parameters according to the population assessed

Considering the results of the current study, there is no unified explanation for Dm, such as in Tc. Muscle atrophy increases Dm in the rigorously controlled physical inactivity model (Pišot et al., 2008; Šimunič et al., 2019), whereas vigorous high-speed exercise decreases Dm in both young (Zubac and Šimunič, 2017) and senior people (Zubac et al., 2019). Only four different aged populations were assessed for Dm. In proximal postural muscles such as VL, the asymptomatic population showed lower Dm than nursing home residents, and the highest Dm was found in patients with knee osteoarthritis, confirming lower physical activity levels or inactivity-induced atrophy paradigm. However, it is not so in GM and BF. In distal postural GM muscle, nursing home participants and peripheral arterial disease patients have far lower Dm than asymptomatic participants, while patients with knee osteoarthritis have the highest Dm, similarly as in the VL muscle. As peripheral arterial disease patients have a much different muscle composition than the asymptomatic population due to ischemic conditions, it is expected to differ, considering muscle contractile properties as well. However, the lowest Dm in nursing home residents indicates that immobility is associated with lower Dm (Fabiani et al., 2021). Lower Dm of postural muscles in less mobile populations could be attributed to accompanying adaptation of the muscle tissue to disuse and aging, e.g., lower muscle (contractile) quality due to infiltration of fat deposits within the muscle tissue, impaired neuro-muscular junction yielding impaired excitation–contraction coupling, and fibrosis due to the excessive formation of fibrous bands of scar tissue in between muscle fibers (Tieland et al., 2018). It is also known that older people have lower elasticity of muscle fibers (Ochala et al., 2007) that could arise due to selective atrophy, and loss and remodeling of muscle fibers toward type I, which are less elastic than the fast-twitch fibers (Mutungi and Ranatunga, 1996).

In contrast, it seems that in non-postural muscles such as BF, trends appear to be different. As the muscle is not regularly involved in habitual activities, it remains unclear whether it is already changed in the older asymptomatic population when compared to the younger counterparts. So far, the mechanisms of such distinctions have remained unclear. Patients with knee osteoarthritis have by far the lowest Dm in comparison to nursing home residents and the asymptomatic group. Suggesting additional mechanisms for Dm regulation in non-postural muscles in the knee osteoarthritis population could be increased chronic inflammation, joint degradation, long-persistent pain, changed gait patterns, or morphological changes inducing arthrogenic muscle inhibition and lower muscle response to electrical stimuli (Fink et al., 2007; Conroy et al., 2012; Ghazwan et al., 2022).

### 4.4 Limitations and future directions

At the end, we must mention some limitations to the current literature review. The heterogeneity of included studies, considering the sample health status, different muscles assessed, and parameters reported, made it difficult to conduct a meta-analysis, and precisely quantify and compare obtained results. However, the present review shows that TMG can be safely used in older adults in a variety of settings. Briefly, it can be used to evaluate muscle contractile properties in both asymptomatic subjects and those diagnosed with musculoskeletal diseases. A part of the mentioned heterogeneity limitation is the age range of samples of included studies as the youngest participant’s mean age was 61.12 ± 5.34 years, and the oldest sample’s mean age was 86.74 ± 7.88 years, resulting in a 25-year age range. This makes it challenging to directly compare the findings as muscular changes occur at an increasing rate after the age of 65. However, having in mind that TMG Tc declines with age, the increased decline was confirmed only in aerobic master athletes, not in anaerobic master athletes and non-athletes (Šimunič et al., 2018). Nevertheless, we believe that TMG could be a promising diagnostic tool for different musculoskeletal diseases, especially in sarcopenia assessment. Additionally, further studies should consider using the TMG device as a diagnostic tool in defining muscle quality in the elderly as it has not been used for this purpose to date.

## 5 Conclusion

The review sought to establish reference values for the main TMG parameters measured in older adults, emphasizing the importance of considering age, physical activity, and comorbidities when interpreting TMG results. The studies included in this review provide important insights into the potential of TMG use as a non-invasive, reliable, objective, and responsive tool for assessing muscle function in older adults. The use of TMG in older adults shows potential to contribute to the early detection of muscle dysfunction and to monitor interventions aimed at preventing or slowing down muscle decline in this population. However, further research is needed to determine its clinical value.

## Data Availability

The original contributions presented in the study are included in the article/supplementary materials; further inquiries can be directed to the corresponding author.
